# A Clinical Text-Book of Medical Diagnosis

**Published:** 1892-05

**Authors:** 


A CLINICAL TEXT-BOOK OF MEDICAL DIAGNOSIS FOR PHYSICIANS AND STUDENTS, based on the most recent methods of examination. By Oswald Vierordt, M. D. Authorized translation from the second improved and enlarged German edition, with additions by Francis H. Stuart, A. M., M. D., with one hundred and seventy-eight illustrations. Philadelphia: W. B. Saunders, 913 Walnut Street, 1891. 'Price, cloth, $4; sheep, $5, net.

This book is incomparably the best work on Diagnosis that we have yet seen. We have spent some weeks in a careful perusal of it and are much impressed with its comprehensive character, its thoroughness, and the advanced methods it teaches for examination of the patient.

The work is divided into three parts, comprising eight chapters, and an appendix. Part First contains the introduction and some general directions. Part Second is devoted to a general examination; while Part Third gives the particular examination of the various organs. In this part are five chapters which give the methods of examination respectively of the Respiratory Apparatus, the Circulatory Apparatus, the Digestive Apparatus, the Urinary Apparatus, and of the Nervous System.

Of these the most remarkable are the examination of the heart, the examination of the kidneys, and the examination of the nervous system.

The examination of the heart is very thorough, and aided by diagrams which give graphic representations of the heart sounds under all pathological conditions, enable the student to learn the heart sounds in the absence of a reliable teacher.

The chapter upon the examination of the nervous system opens with a very clear description of the anatomy and the normal and pathological physiology of the brain illustrated by diagrams with shaded regions after the manner of a map showing the situations of the various "tracts," and origins of systems of nerves.

Too much commendation cannot be given this valuable treatise, which ought to be a text- book in every college and is destined sooner or later to be in the hands of every progressive physician. W. M. J.

